# The Influence of Physical and Physiological Cues on Atomic Force Microscopy-Based Cell Stiffness Assessment

**DOI:** 10.1371/journal.pone.0077384

**Published:** 2013-10-23

**Authors:** Yu-Wei Chiou, Hsiu-Kuan Lin, Ming-Jer Tang, Hsi-Hui Lin, Ming-Long Yeh

**Affiliations:** 1 Department of Biomedical Engineering, National Cheng Kung University, Tainan, Taiwan; 2 Department of Physiology, National Cheng Kung University, Tainan, Taiwan; LAAS-CNRS, France

## Abstract

Atomic force microscopy provides a novel technique for differentiating the mechanical properties of various cell types. Cell elasticity is abundantly used to represent the structural strength of cells in different conditions. In this study, we are interested in whether physical or physiological cues affect cell elasticity in Atomic force microscopy (AFM)-based assessments. The physical cues include the geometry of the AFM tips, the indenting force and the operating temperature of the AFM. All of these cues show a significant influence on the cell elasticity assessment. Sharp AFM tips create a two-fold increase in the value of the effective Young’s modulus (E_eff_) relative to that of the blunt tips. Higher indenting force at the same loading rate generates higher estimated cell elasticity. Increasing the operation temperature of the AFM leads to decreases in the cell stiffness because the structure of actin filaments becomes disorganized. The physiological cues include the presence of fetal bovine serum or extracellular matrix-coated surfaces, the culture passage number, and the culture density. Both fetal bovine serum and the extracellular matrix are critical for cells to maintain the integrity of actin filaments and consequently exhibit higher elasticity. Unlike primary cells, mouse kidney progenitor cells can be passaged and maintain their morphology and elasticity for a very long period without a senescence phenotype. Finally, cell elasticity increases with increasing culture density only in MDCK epithelial cells. In summary, for researchers who use AFM to assess cell elasticity, our results provide basic and significant information about the suitable selection of physical and physiological cues.

## Introduction

The cytoskeleton is a salient constituent of a cell. By forming as a hierarchical meshwork, the cytoskeleton provides the structural stabilization of the cell. Cytoplasmic enzymes, proteins, and the cytoskeleton are involved in the coordination of several signal pathways. Such interplays help a cell to accommodate to external environment stimuli by assembling or disassembling the cytoskeleton instantaneously. Consequently, several cell behaviors are regulated by the cytoskeleton, including cell shape determination [1], migration [2], proliferation [3], adhesion [4], and others. Microfilaments, intermediate filaments, and microtubules are three major components of the cytoskeleton. Hindering the formation of those cytoskeleton filaments by inhibitors leads to decreased cell elasticity [5]. The actin filament is suggested to be the most significant cytoskeleton component for modulating the mechanical properties of cells [6], [7]. *In vivo*, microfilaments are relevant to many cell functions, such as differentiation, motility, maintenance of cell junction formation, and changes in cell morphology, [8]–[10]. A cell needs to form proper contacts with the extracellular matrix (ECM). Therefore, integrin-mediated adhesion signals are generated via focal adhesion kinase (FAK) and subsequently trigger the formation of the focal adhesion complex (FAC). Simultaneously, actin polymerization is initiated, which consequently reinforces the strength of FACs. Such processes, then, form a positive feedback loop for modulating cell behaviors [11]–[15]. The relevant system forms by actin polymerization and myosin II activity that is controlled by the small GTPase RhoA and its downstream ROCK. Moreover, extensive studies have demonstrated that the actomyosin tension formed by actin polymerization regulates the morphology and function of the nucleus via the linker of nucleoskeleton and cytoskeleton (LINC) complex [16]–[19]. Actin filaments are also important in the formation and maintenance of cell-cell interactions. Indeed, a sturdy adherens junction requires the intracellular recruitment of actin filaments to adhere to the cross-linking complex of E-cadherins with α, β, p120, plakoglobin catenin, and others [20]. Inhibition of actomyosin tension leads to disruption of the cell junction [21]. Hence, it is plausible for us to understand the mechanical properties of the cell through the perspective of microfilament assembly.

**Figure 1:**
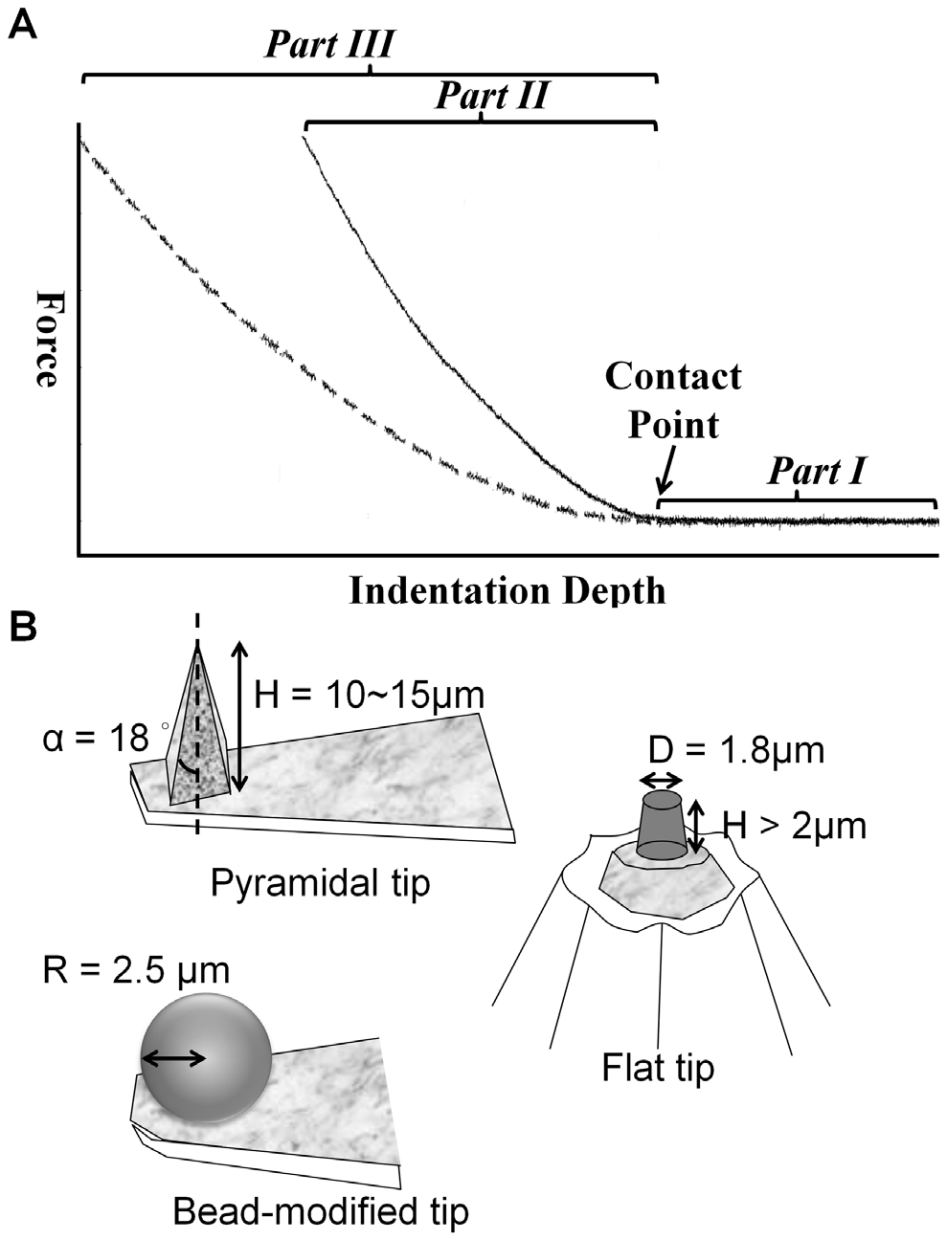
Representative force-indentation curves from AFM and sketches of the tip geometry. (A) The dotted and solid lines represent distinct traces of the approach of the AFM tip, as measured from samples with different physical properties. Initially, the AFM tip was located at the designed position over the sample. As the AFM tip start to approach the sample, there was no interaction force (*Part I*). After the AFM tip contacted with the sample at the contact point (shown by black arrow), further indentation generates the indentation depth. Constant force generates a greater indentation depth on the softer cell (*Part III*) than on the stiffer cell (*Part II*). (B) Tips with three different geometries were used in this study.

In cell mechanics studies, atomic force microscopy (AFM) has become a powerful instrument, which allows researchers to assess the delicate topography and biomechanical properties of living eukaryotic cells [22]–[25]. Compared with other instruments, AFM has the advantage of high resolution, measurability in a natural aqueous environment, and easy sample preparation [26]. In general, we apply an appropriate force through the AFM tip to indent a cell first and then generate a comprehensive deformation of the cell. To understand the relation between applied force and cell deformation, we must unveil the information that is hidden inside the force-displacement curve. As shown in Fig. 1, the force-displacement curve, which is recorded for the entire contact history, could be further analyzed by Hooke’s law, contact mechanics, viscoelasticity or other relevant formulae. By selecting the most appropriate method, the mechanical properties of living eukaryotic cells can be determined [27]–[30].

Studies of accumulated cell elasticity via AFM have been reported. However, even for well-studied NIH3T3 cells, the effective Young’s modulus (E_eff_) of these AFM studies spanned from less than 1 kPa to 7 kPa [31]–[34]. Such variations might be due to dissimilar types of tip, uneven temperature selection and the variety of culturing conditions [35], [36]. Moreover, the experimental conditions of cells (culturing medium, culture density, and culture passage number) and the AFM operation parameters (temperature, applied indenting force, loading rate, and the shape of the AFM tips) are seldom compared [31], [37]. Because the conditions and parameters of earlier studies have not been sufficiently described, the method of the current study is crucial. In this study, we try to evaluate the elasticity of cells while varying the assessment parameters and culturing conditions. The aim of this investigation is to identify the physiological and physical factors that influence AFM-based assessments, either independently or synergistically.

## Materials and Methods

### Cell Line and Cell Culture

NIH3T3 and 7-4 (Ha-Ras^V12^ oncogene transformed) cells were kindly gifts from Dr H. S. Liu (Department of Microbiology and Immunology, National Cheng Kung University, Taiwan). Madin-Darby canine kidney (MDCK) cells were purchased from American Type Culture Collection (Manassas, VA) [38]. These cell lines were maintained in Dulbecco’s modified Eagle’s medium (DMEM, Sigma-Aldrich, St. Louis, MO) supplemented with 10% fetal bovine serum (Invitrogen, Carlsbad, CA), 2 mM L-glutamine, 100 IU/ml penicillin, and 100 µg/ml streptomycin (Sigma-Aldrich, St. Louis, MO). The origin and development of the mouse kidney progenitor cells (MKPC) have been described in the previous study [39]. MKPC at passages around 20^th^, 30^th^, 50^th^, and 90^th^ were used and maintained in DMEM supplemented with 10% calf serum, 2 mM L-glutamine, 100 IU/ml penicillin, and 100 µg/ml streptomycin. All the cell lines were regularly cultured at 37°C in a 5% CO_2_, humidified incubator.

### Adherent Substrate Preparation

Cells were plated on Type I collagen (COL I, 50 µg/ml, BD Biosciences PharMingen, San Jose, CA)-coated glass slides at the density of <50 cells/mm^2^ in growth medium for most AFM assessments in this study, besides the specific conditions. To examine the E_eff_ of NIH3T3 cells adhered to various substrates, glass slides were coated with the following: COL I (50 µg/ml), Type IV collagen (COL IV, 100 µg/ml), fibronectin (FN, 10 µg/ml), gelatin (GEL, 1 mg/ml), and poly L-Lysine (PLL, 100 µg/ml). To check the effect of ECM concentration on the cell stiffness, cells were plated onto glass slides coated with Col I at the concentration of 50, 100, or 1000 µg/ml. To check the effect of substrate compliance on cell stiffness, cells were plated onto culture dish, collagen gel-coated culture dish, and collagen gel (1 mg/ml) with the E_eff_ about 100 Pa [40], respectively.

### Culturing Parameters

To test the effect of FBS on cell stiffness, cells were cultured in DMEM or CO_2_-independent medium (CO_2_-IDM) (Gibco) supplemented with or without 10% FBS. However, cells cultured in CO_2_-IDM should be maintained at 37°C in a humidified incubator. To test the effect of indentation temperature on cell stiffness, BioCell® (JPK Instruments AG, Germany) was used to ensure AFM operation at 31°C and 37°C. Thermocouple (model: st-54, Suntex, Taiwan) was used to measure the temperature of solution under exposure to the light of the microscope. To test the effect of culture density on cell stiffness, cells were cultured at the density of 5, 50, 500, or 900 cells/mm^2^ on COL I-coated dish for 16 h.

### AFM System

The JPK NanoWizard® II AFM system installed on top of an inverted light microscope (Zeiss Axio Observer) on a an anti-vibration table and enclosed by a custom-built anti-noise box. BioCell® (JPK Instruments AG, Germany) was used to ensure AFM operation at 37°C.

### AFM Cantilevers and Measurement Parameters

**Figure 2:**
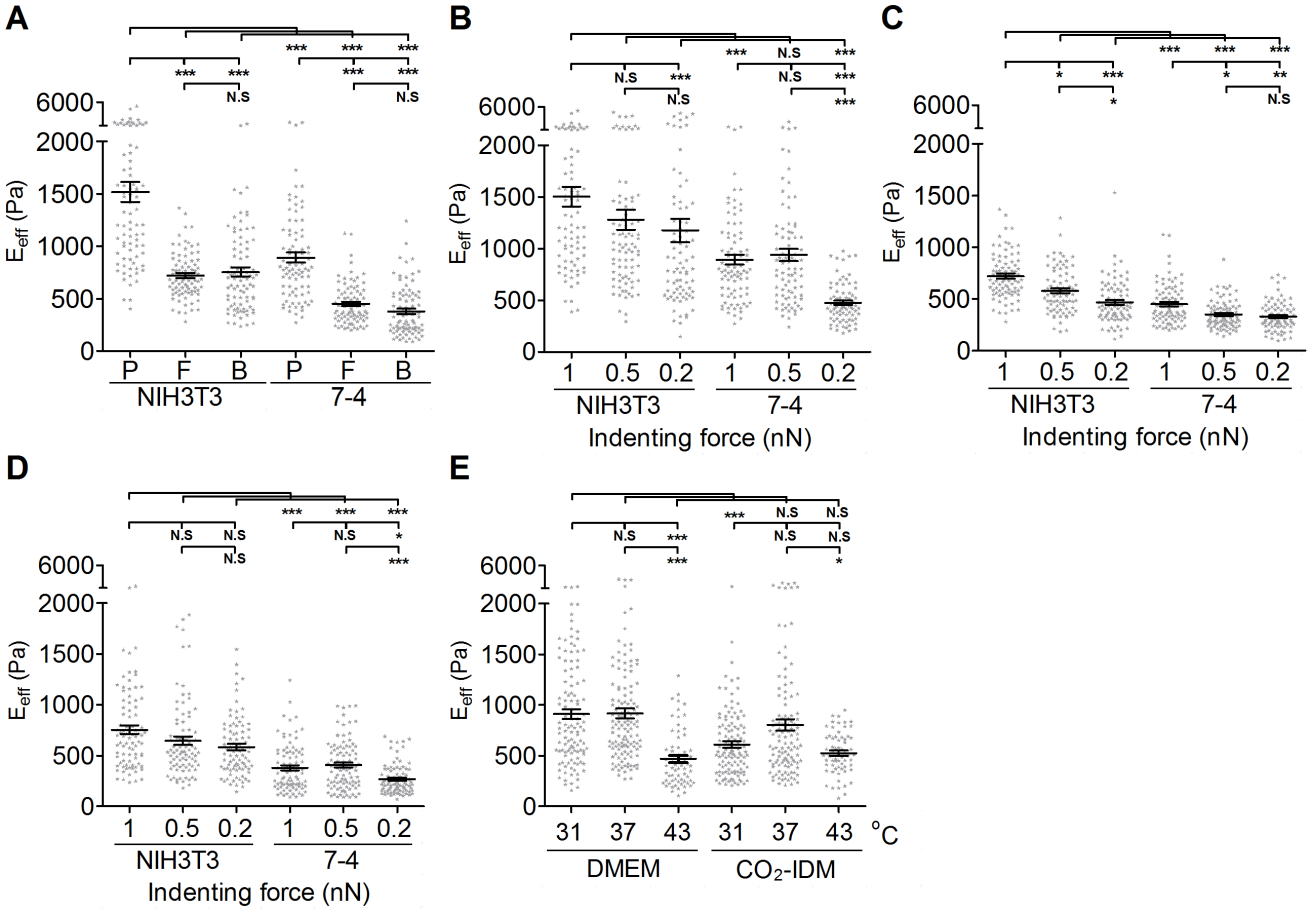
The effect of AFM tip shape, indenting force at the same loading rate, and operating temperature on the effective Young’s modulus of cells. To determine the effect of the AFM tips, (A) NIH3T3 cells and 7-4 cells were plated on type I collagen (COL I)-coated glass slides overnight. The effective Young's moduli (E_eff_) of cells were measured by Bio-AFM with a pyramidal tip (labeled as P in (A)), flat tip (labeled as F in (A)), and 5 µm-bead-modified tip (labeled as B in (A)) with a 1 nN indenting force at 1 µm/sec approach velocity. To determine the effect of the indenting force at the same loading rate, cells were plated on COL I-coated glass slides overnight. The E_eff_ of cells were measured by Bio-AFM with a (P) pyramidal tip, (F) flat tip, and (B) 5 µm-bead-modified tip with different indenting force (0.2, 0.5 or 1 nN). To evaluate the effect of operating temperatures, (E) NIH3T3 cells were plated on COL I-coated glass slides and cultured in DMEM at 31°C, 37°C, and 43°C and in CO_2_-independent medium (CO_2_-IDM) at 31°C and 37°C. The results were expressed as the mean ± SEM by scatter dot plot. Gray symbols represent the detailed experimental data. ***p<0.001; **p<0.01; *p<0.05; N.S, no significance.

To investigate the E_eff_ of cells obtained by AFM tips with different shapes, a 5 µm (in diameter) polystyrene bead-modified tip-less cantilever (ARROW-TL1-50, NanoWorld, US), a flat tip cantilever with approximately 1.8 µm in diameter of plateau (PL2-CONT-10, NanoWorld, US), and a pyramid tip cantilever with less than 10 nm nominal radius in the apex (T1L450B, NANOSENSORS, US) were utilized (Fig. 2A). The nominal spring constants of all cantilevers ranged from 0.02 to 0.08 N/m. The spring constants of all cantilevers were calibrated with the thermo noise method prior to each experiment. The applied forces in this study were set to 0.2 nN, 0.5 nN, and 1 nN, respectively, and the corresponding displacements on cells were recorded simultaneously. The approaching and retracting rates of cantilever were set at 1 µm/sec. Thus, all applied forces approached the cell at the same loading rate (1 nN/s). In order to rule out the data bias due to the contamination of the AFM tip, polyacrylamide (PA) gel was prepared as a reference substrate. All tips will indent the PA gel before and after cell indentation assessments to evaluate for any variation in the value. The E_eff_ of PA gel obtained from pre-cell-indentation should be consistent in those from post-cell-indentation.

### Topographic Images by AFM

The AFM images were all conducted by pyramidal tip (T1L450B, NANOSENSORS, US) in contact mode with 200 µm/sec scanning velocity. The scanning force (generally less than 1 nN) and the operation gains were adjusted according to the condition of cells instantly.

### Data Analysis

All indentation depth curves were calculated with the JPK package software, which is based on the Hertz model [28], [29]. The E_eff_ obtained for the bead-modified, pyramidal, and flat tips were calculated using Eq 1, 2, and 3, respectively:
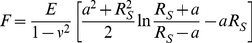
(1)

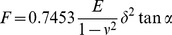
(2)

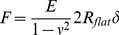
(3)Where *F* represents force, *E* represents E_eff_, *ν* represents Poisson’s ratio (0.5 in this study), *δ* represents the indentation (tip sample separation), *a* represents the radius of the contact circle, *R_flat_* represents the plateau radius of the flat tip (0.9 µm in this study), *α* represents the half open angle of the pyramidal tip (18° in this study) and *R_S_* represents the radius of the bead-modified tip (2.5 µm in this study).

Each cell was indented once on the top center of nucleus. For each experiment, more than 60 cells were measured, these experiments were repeated at least twice. Graphpad Prism (Graphpad Software, San Diego, CA) was used to calculate and plot mean and standard error of the mean (SEM) of measured quantities. The results were expressed by scatter dot plot with mean ± SEM. To ascertain whether the groups follow the Gaussian distribution, we administered the Kolmogorov-Smirnov test on all the groups. With this test, none of the data groups in this study was shown to display Gaussian distribution. Consequently, we applied the Kruskal-Wallis test and Dunn's multiple comparison test to analyze the data.

### Immunofluorescence Staining

**Figure 3:**
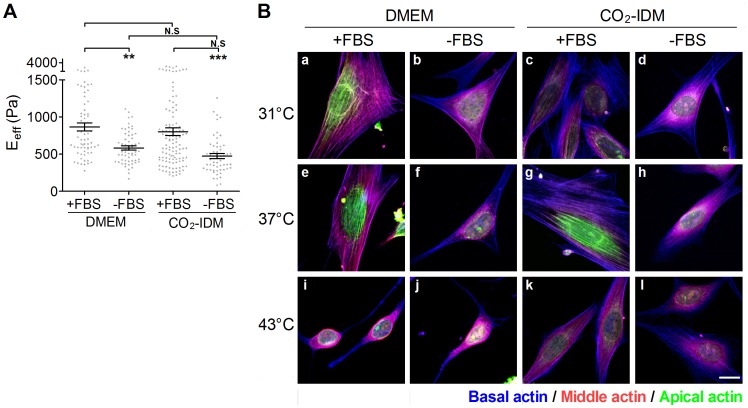
The effect of culture conditions on the effective Young’s moduli of cells. NIH3T3 cells were plated onto COL I-coated glass slides and culured in DMEM (or CO_2_-IDM) supplemented with or without FBS overnight. The incubating temperatures were set at room temperature (31°C), 37°C, and 43°C. (A) The cells were cultured in DMEM or CO_2_-IDM supplemented with or without 10% FBS at 37°C, respectively. The effective Young’s moduli (E_eff_) of cells were assessed by the Bio-AFM. The results are showed in scatter dot plot by mean with standard error (SE). **p<0.01; *p<0.05; N.S., no significance. (B) The representative Max XY projection images of cells cultured in various conditions. Actin cap fibers in the apical region of the cell were re-colored green, the stress fibers in the middle region of cell were colored red, and the stress fibers in the the basal region of cell were re-colored blue. (Scale bar = 10 µm).

**Figure 4:**
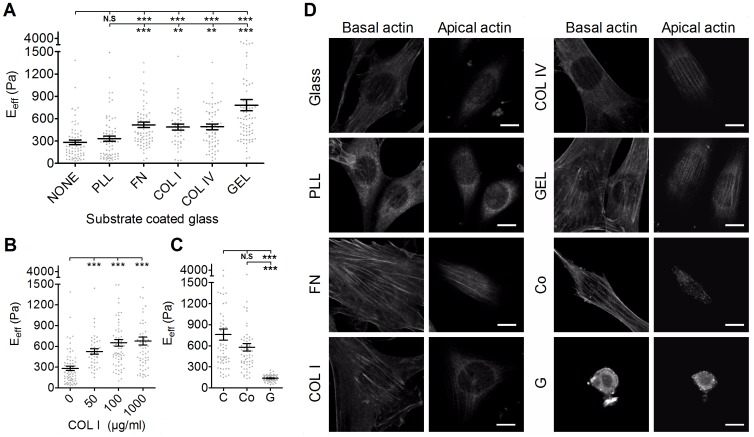
The effect of various substrates on the effective Young’s moduli of cells. (A) NIH3T3 cells were plated onto glass slides coated with various substrates (PLL, poly-L-Lysine; FN, fibronectin; COL I, type I collagen; COL IV, type IV collagen; GEL; gelatin) overnight. (B) To evaluate the effect of substrate concentration on the effective Young’s moduli (E_eff_) of cells, cells were plated onto glass slides coated with COL I of various concentrations (50, 100, or 1000 µg/ml). (C) To evaluate the effect of substrate compliance, cells were plated onto culture dish (C), collagen gel-coated dish (Co), and collagen gel (G). The E_eff_ of cells were assessed by Bio-AFM. The results were expressed as the mean ± SEM by scatter dot plot. Gray symbols represent the detailed experimental data. ***p<0.001; **p<0.01; N.S, no significance. (D) Organization of actin filament in the apical actin and basal actin in NIH3T3 cells plated on different substrate (glass, PLL, FN, COL I, COL IV, GEL, Co, G). (Scale bar = 10 µm).

Cells grown on different culturing condition were fixed in 4% paraformaldehyde for at least an hour and then washed twice with phosphate-buffered saline (PBS). Cells were permeabilized in PBS containing 0.1% Triton X-100 (Sigma-Aldrich, St. Louis, USA) in PBS and then blocked with SuperBlock buffer (Thermo Scientific, Rockford, IL) for an hour. Cells were incubated with primary antibody for α-tubulin at 4°C overnight. After extensively washing with PBS, cells were incubated with secondary antibody conjugated with Alexa 488 (Invitrogen, Carlsbad, CA), phalloidin-TRITC (Sigma-Aldrich, St. Louis, MO) and Hoechest 33258 (10 µg/ml) for an hour at room temperature. Finally, immunocomplexes were visualized under the confocal microscopy (Olympus FV-1000, Tokyo, Japan) or epifluorescence microscopy (Nikon Eclipse Ti, Tokyo, Japan). In order to examine the relationship between the spatial distribution of cytoskeleton and cell elasticity, immunofluorescence observation was conducted under confocal microscope. The imaging was performed from sequential z-series scans with the FluoView™ FV1000 confocal microscope (Olympus, Tokyo, Japan) at high zoom (2.0–5.0) with a 60 × water immersion lens, NA 1.35 (Uplsapo). Two-dimensional (2D) maximium (Max) intensity projection images with “z projection’ function (for Fig. 3B) via the ImageJ software (NIH) was conducted to reconstruct the whole architechture of cell. In some cases, we manifested the importance of the actin cap (the apical actin filaments across the nucleus) on elastic moduli of cells cultured on different condition (for Fig. 4 and Fig. S2). More than 30 cells from each culture condition were checked and the representative images were showed in each figure.

## Results

### Effect of AFM Tip Shapes, Indenting Forces, and Operating Temperature on Cell Stiffness

To investigate the effect of AFM tip shapes on the measurement of cell stiffness, we used AFM tips with different shapes, as described in the Materials and Methods as well as in Fig. 1B, to indent cells. NIH3T3 and 7-4 cells were seeded at a subconfluent density (<50 cells/mm^2^) and cultured in growth medium for 24 hours. As shown in Fig. 2A, the E_eff_ of a cell measured using the pyramidal tip is significantly greater than those obtained using the flat tip or the bead-modified tip, regardless of cell types. There is no significant difference for the E_eff_ measured using the flat tip and the bead-modified tip. Moreover, NIH3T3 cells display an almost two-fold increase in the E_eff_ over that of 7-4 cells, irrespective of the AFM tip shape. To investigate the effect of indenting force with the same loading rate, we changed the applied force (1 nN, 0.5 nN, and 0.2 nN) with a constant approaching/retracting rate. In general, the lower values of the E_eff_ were associated with lower indenting force for both NIH3T3 and 7-4 cells. The E_eff_ of cells measured using pyramidal tip indentation was significant reduced only at the indenting force of 0.2 nN-µm/sec for both NIH3T3 and 7-4 cells (1 vs. 0.2, p<0.001 in NIH3T3; 1 vs. 0.2, p<0.001 in 7-4) (Fig. 2B). When the flat tip was used, the E_eff_ of cells decreased significantly with decreased indenting force at the same loading rate for both NIH3T3 and 7-4 cells (Fig. 2C) (1 vs. 0.5, p<0.05; 1 vs. 0.2, p<0.001 in NIH3T3; 1 vs. 0.5, p<0.05, 1 vs. 0.2, p<0.01 in 7-4). There were no significant differences in the E_eff_ of NIH3T3 cells that were measured with indentations from the 5-µm-bead-modified tip at different indenting force at the same loading rate. In 7-4 cells, only the indenting force at 0.2 nN resulted in a significant but slight decrease in the E_eff_ (Fig. 2D) (p<0.05). Tables 1 and 2 summarize all the details of the values for the E_eff_ of NIH3T3 and 7-4 cells measured using different AFM tips and indenting force at the same loading rates. Temperature plays a very important role in regulating the protein/enzyme activities that are correlated with cell behavior. To evaluate the effect of operating temperature on the measurement of cell stiffness, we set the operating temperature at 31°C, 37°C, and 43°C with the aid of Bio-cell. NIH3T3 cells were seeded on COL I-coated glass coverslips in the growth medium or in CO_2_-IDM supplemented with 10% FBS for 24 h before indentation with the AFM beaded tip. The indenting force was 1 nN with 1 µm/sec approach velocity. As shown in Fig. 2E, the E_eff_ of cells cultured in growth medium that was measured at 43°C was statistically lower than those measured at 37°C and 31°C (37°C vs. 43°C, p<0.001; 31°C vs. 43°C, p<0.001) (Fig. 2E). There were no significant differences for the E_eff_ of cells obtained at 37°C and 31°C in growth medium or CO_2_-IDM medium. Moreover, cells cultured in CO_2_-IDM were significant softer than cells cultured in normal growth medium (p<0.05). These results suggested that the constitution of the culture medium might play important roles in regulating the function and mechanical properties of cells.

**Table 1:** The effective Young’s moduli of NIH3T3 cells measured at different indenting force at the same loading rate.

	0.2 nN	0.5 nN	1 nN
**Pyramidal**	1189.0±113.7Pa (80)	1291.3±97.5 Pa (86)	1518.6±95.5 Pa (82)
**Flat**	467.6±22.8Pa (84)	578.4±23.5 Pa (82)	720.8±23.8 Pa (84)
**5 µm-Bead**	583.6±31.5Pa (86)	647.2±40.0 Pa (89)	753.1±42.7 Pa (86)

Each value represents the mean ± standard error (cell number for analysis).

**Table 2:** The effective Young’s moduli of 7-4 cells measured at different indenting force at the same loading rate.

	0.2 nN	0.5 nN	1 nN
**Pyramidal**	476.1±21.9 Pa (78)	909.3±59.6 Pa (83)	892.6±48.4 Pa (85)
**Flat**	329.4±15.0 Pa (83)	348.9±14.8 Pa (83)	450.6±21.3 Pa (84)
**5 µm-Bead**	267.2±15.7 Pa (87)	407.2±23.9 Pa (97)	379.9±25.4 Pa (86)

Each value represents the mean ± standard error (cell number for analysis).

### Effect of Culturing Conditions on the Cell Stiffness

To evaluate the effect of the culturing medium on the cell stiffness, NIH3T3 cells were cultured in DMEM or CO_2_-IDM supplemented with or without 10% FBS at 37°C, respectively. FBS-supplemented medium significantly increased the E_eff_ of cells (p<0.01) (Fig. 3A), regardless of the type of medium. The acrhitecture of actin filaments were displayed by the Max intensity XY projection images as shown in Fig. 3B. NIH3T3 cells cultured in DMEM and CO_2_-IDM supplemented with FBS showed the most robust structures with actin cap formation (Fig. 3B, e and g).

### Effect of the Adhering Substrate on Cell Stiffness

In general, the E_eff_ of NIH3T3 cells plated on FN, COL I, COL IV, and GEL was significantly higher than in cell plated on glass or PLL (Fig. 4A). There was no significant difference in the E_eff_ for cells plated on PLL or glass. Cells plated on GEL showed the highest E_eff_. Cells plated on COL I were stiffer than cells plated on glass, irrespective of the coating concentration (Fig. 4B). However, cells plated on collagen gel (G), the soft gel form, were significant softer than cells plated on normal culture dish (C) or collagen gel coated-dish (Co). We then asked whether NIH3T3 cells differentially formed a perinuclear actin cap when plated on different substrate. Confocal fluoresence microscopy was used to examine the organization of actin filaments in NIH3T3 cells. The same cells were first scanned at small increments between basal section and apical section as to not miss actin structure surrounding the nucleus. As shown in Fig. 4C, actin filaments at the apical surface of the nucleus in NIH3T3 cells plated on FN-, COL I-, COL IV-, GEL-, and collagen gel-coated dish formed thick bundles that were mostly parallel to one another in the cap and globally parallel to the direction of the long axis of the nucleus. NIH3T3 cells plated on glass slides or PLL-coated glass slides exhibited weak and less actin filament in the cap. However, remarkably, all examined NIH3T3 cells plated on collagen gel were devoid of organized actin filament structure above the nucleus and in the basal region. These results suggest that both the chemical composition and physical properties of ECM affect the organization of actin filament, and consequently the cell elasticity.

### Effect of Culture Passage Number on Cell Stiffness

**Figure 5:**
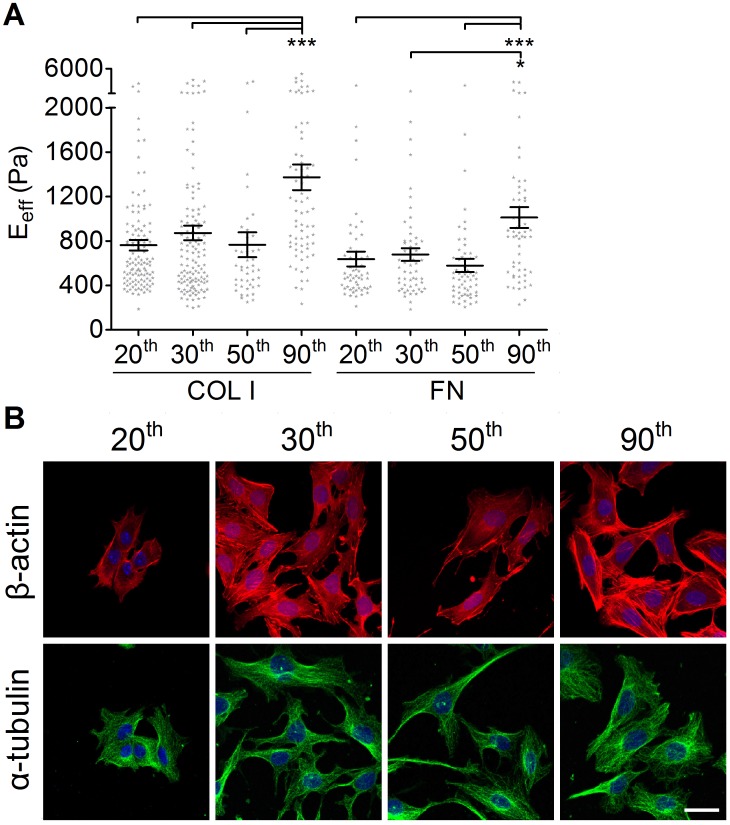
The effect of culture passage number on the effective Young’s moduli of MKPC. (A) MKPC at the 20^th^, 30^th^, 50^th^, and 90^th^ passages were plated onto type I collagen- or fibronectin-coated glass slides overnight. The results were expressed as the mean ± SEM by scatter dot plot. Gray symbols represent the detailed experimental data. (***p<0.001; *p<0.05) (B) MKPC at the 20^th^, 30^th^, 50^th^, and 90^th^ passages were stained and represented as the maximal section of confocal immunofluorescence images of β-actin (red) and α-tubulin (green). (Scale bar = 10 µm).

Comparing the E_eff_ for MKPC at different culture passage numbers, we found that there were no significant differences for cells at passages 20^th^, 30^th^, and 50^th^ (Fig. 5A). However, the E_eff_ of MKPC increased significantly at its 90^th^ passage. In addition, MKPC plated on either COL I or FN exhibited similar cell stiffness and only became significantly stiffer at the highest culture passage number. To understand the possible factors that were related to the increase of rigidity, we performed immunostaining for actin and microtubules, which were then visualized using confocal microscopy. As shown in Fig. 5B, the actin fibers of MKPC at passage 90^th^ have more cross-linking and are thicker than those of cells at earlier passages. However, the intensity and organization of the microtubule networks was similar in MKPC at different passages.

### Effect of Culture Density on Cell Stiffness

**Figure 6:**
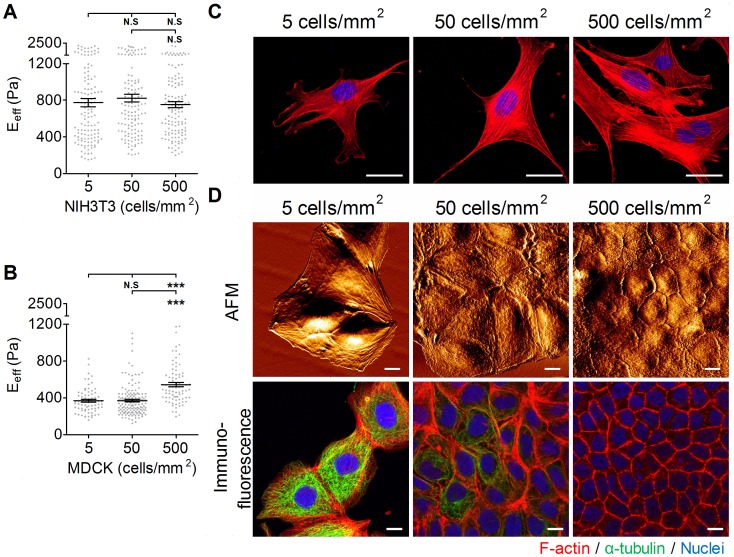
The effect of plating density on the effective Young’s moduli of cells. (A) NIH3T3 cells and (B) MDCK cells were plated onto COL I-coated glass slides at densities of 5, 50, or 500 cells/mm^2^ overnight. The effective Young’s moduli (E_eff_) of cells were assessed by Bio-AFM. The results were expressed as the mean ± SEM by scatter dot plot. Gray symbols represent the detailed experimental data. (***p<0.001; N.S, no significance) (C) NIH3T3 cells were plated onto COL I-coated glass slides at densities of 5, 50, or 500 cells/mm^2^. The immunofluorescence results are represented as F-actin (red) and nuclei (blue). (D) AFM surface topological images in living MDCK cells and confocal immunofluorescence images of F-actin (red), α-tubulin (green), and the nucleus (blue) in stained MDCK cells that were cultured at densities of 5, 50, or 500 cells/mm^2^, respectively. (Scale bar = 10 µm).

To study whether cell stiffness is affected by culture density, cells were plated at different densities that mimicked the degree of confluence of the cultures: ∼5 cells/mm^2^ for sparse culture, ∼50 cells/mm^2^ for sub-confluent culture, ∼500 cells/mm^2^ for confluent density, and ∼900 cells/mm^2^ for over-confluent density. We use two different cell types: NIH3T3 cells, in which the mesenchyme exists mostly without cell-cell interaction; and MDCK cells, which are noted for having elaborate cell-cell interactions. As shown in Fig. 6A, the E_eff_ of the NIH3T3 cell did not change significantly with the culture density. Similarly, confocal microscopy images of NIH3T3 showed that cells displayed many prominent F-actin stress fibers, regardless of the culture density (Fig. 6C). The E_eff_ of MDCK cells plated at the sparse density was statistically indistinguishable from the E_eff_ of MDCK cells plated at the sub-confluent density. However, there was a significant increase (95%) in the E_eff_ of MDCK cells plated at the confluent density (Fig. 6B). AFM images of MDCK cells revealed that the integrity of cell-cell junctions was enhanced by increasing the culture density (Fig. 6D). In the sparse and sub-confluent densities, MDCK cells exhibited less or unapparent cell-cell junctions, and furthermore, the cell elasticity of MDCK cells did not increase with the number of surrounding cells (see Fig. S1A). At the confluent density, the scanned cell-cell junctions appeared dense with a continuous narrow line structure. Immunofluorescence confocal images of MDCK cells confirmed that the maturation of cell-cell junctions correlated to cell confluence (Fig. 6E). At the sparse and sub-confluent densities, the randomly distributed cells formed no or weak and discontinuous junctional actin. At the confluent density, cells formed a compact monolayer with strong and continuous junctional actin. The X–Z section images revealed that more extensive microtubule networks were observed in cells at the sparse and sub-confluent densities than at the confluent density (Fig. S1B). Interestingly, the very elaborate microtubules gradually degenerated when cells reached the confluent stage.

## Discussion

The tip geometry is a salient factor in AFM assessment; the geometry not only governs the distribution of the stress concentration but also determines the contact conditions between the AFM tip and the objective. According to the geometry of the AFM tip, many formulae were derived from the Hertz or non-Hertz model that allowed AFM users to determine the mechanical properties of the cell more precisely [28], [41]. In this study, we assessed the E_eff_ of cells using three different tips: pyramidal, flat, and bead-modified tips. Apparently, the E_eff_ of cells measured using pyramidal tip indentation was significantly higher than those of cells measured using indentations with the flat tip and the bead-modified tip. Moreover, the distribution of the rigidity measurements using the pyramidal tip was also noticeably broader than those for the other two tips. This result might be due to the geometric differences of the AFM tips. The pyramidal tip is 10–15 µm in height and less than 10 nm at the apex. The flat tip is 1.8 µm in width, and the bead-modified tip is 5 µm in diameter. The sharpness of the apical point of the pyramidal tip might result in a poorly defined contact area, diverse indentation depth, and incorrect contact point prediction, which in turn leads to overestimation of the E_eff_ and large standard deviation. Using AFM and confocal microscopy, Harris et al. also found that the predicted contact point and indentation depth of a bead-modified tip for AFM could be verified by confocal images, but those of the pyramidal tip could not [35]. The AFM topological scanning images revealed that many well-aligned thick fibers (∼400 nm in width) interlaced to form a meshwork-like structure on the top of a NIH3T3 cell nucleus (Fig. S2). When using the pyramidal tip to indent cell, the target of the tiny apex could be the thick fiber that displayed higher structural strength or the interstice between the fibers that displayed lower structural strength. Such effect would be understandable when a cell possesses a highly organized cytoskeleton and might provide another explanation for the broad distribution in cell stiffness for the pyramidal tip indentations. A previous study indicated that the actin network played a critical role in both the elasticity and viscosity of cells [6]. Compared with the prominent actin filamentous network of NIH3T3 cells, the Ha-Ras^V12^-transformed 7-4 cells displayed a reduced and randomly organized F-actin network (Fig. S2). Therefore, it is plausible that 7-4 cells are softer than NIH3T3 cells and displayed narrowly distributed cell stiffness. In contrast, the large surface contact area facilitated the ability of the flat and bead-modified tips to span multiple filaments during indentation. By distributing the indentation force among multiple strong filaments, the ‘stress sharing’ behavior led to a smaller indentation depth (data not shown), as well as a smaller deviation range in the E_eff_. Therefore, the large range in the E_eff_ distribution for fibroblasts is not surprising [31]–[34].

Cells comprise various components; therefore, the integral outcomes show the material properties of cells as anisotropic, inhomogeneous, and viscoelastic. Such characteristics increase the complexity of analyzing the cell mechanics. To simplify the numerical analysis, many assumptions have been suggested to lessen the complication resulting from cell stiffness. For instance, the Hertz model assumes the cell is an isotropic, homogenous, pure material that undergoes small deformation upon indentation. However, these assumptions might be insufficient for elucidating the cell-indenter contact behavior. Furthermore, the proposed value of Poisson’s ratio remains controversial. Previous studies have shown that the proposed value of Poisson’s ratio for cells ranges from 0.3 to 0.5 [34], [42]; moreover, the value of Poisson’s ratio might depend on cell type, cell structure, and the extracellular environment. To date, no consistent result for Poisson's ratio has been obtained for each cell type. Thus, most AFM studies still adopt the value of 0.5 for Poisson’s ratio. In addition, some studies have shown that the contribution of cell viscosity can be attenuated using an extremely low tip approach velocity (less than 1 µm/sec) [43]–[45]. To diminish the complexity of the numerical analysis, we assumed (1) that all cell-indenter contacts obey the Hertz contact assumptions, (2) a value of Poisson’s ratio of 0.5, and (3) that the approach velocity was set at 1 µm/sec. Given these assumptions, cell elasticity would become dominant when analyzing and comparing the physical properties of cells. We then changed the indenting force and checked the effect on assessing cell elasticity. We found that the E_eff_ of cells were positively correlated with the increased indenting force at the same loading rate in general, regardless of tip shape or cell type (see Fig. 2B–D). Hooke’s law indicates that higher stress causes greater strain. Thus, the higher indenting force elicited a larger cell deformation. Lekka et al. has previously shown that normal cells are always stiffer than other cancerous cell by an indenting force of 2 nN or 10 nN [46]. However, when the Hertz model is used to calculate the cell elasticity, the indenting force and loading rate should be applied conservatively and carefully to obey the assumption of the Hertz model. An improper indenting force and loading rate will damage cells and cause misleading results. For example, using the pyramidal tip with a range of indenting forces from 1 nN to 30 nN was shown to penetrate the eukaryotic cell membrane or even result in cell lysis [47]–[50]. Another study also observed that the sharp tip could penetrate the cell even when applying a very small indenting force at a slow approaching velocity (approximately 1 µm/sec) [50]. The optimal range of indenting force depends on the membrane of the different cell types, the approaching velocity of the AFM tip, and the geometry of the AFM tip. Actually, the force-displacement curve might provide information for evaluating whether the indenting force at the same loading rate is appropriate. When the pyramidal tip was used to indent cells by applying a 2 nN, a noticeable discontinuity in the force trace was frequently observed (Fig. S3). This abrupt peak might be due to the penetration of the sharp tip into the cell membrane. Thus, we set the indenting force to a maximum of 1 nN to prevent the abrupt peak, which might lead an operator to misread the cell stiffness [49], [50]. We determined that NIH3T3 cells always exhibited significant higher E_eff_ than did 7-4 cells, regardless of the indenting force at the same loading rate (Fig. 2B). The high variation in cell stiffness obtained by the pyramidal tip occurred in both NIH3T3 cells and 7-4 cells, irrespective of the indenting force at the same loading rate. This finding might be due to the contact between an extremely small apex and different targets as described previously. At a 0.5 nN-µm/sec indenting force, because the broad distribution of the E_eff_, the pyramidal tip even failed to discriminate NIH3T3 cells from 7-4 cells. When the flat tip was used, the E_eff_ of cells increased significantly as the indenting force increased in both cell types (see Fig. 2C). The E_eff_ of cells also showed a convergent distribution. Surprisingly, the E_eff_ of NIH3T3 cells measured using the bead-modified tip displayed the poorest ability to distinguish cells at any indenting force. The bead-modified tip can indent several cytoskeleton filaments simultaneously because of its large contact area. It is reasonable that the well-organized filaments shared and dissipated the indenting force, diminishing its effect. Taking these data together, we suggest that the flat tip and the bead-modified tip might be better choices for assessing the elasticity of cells. Both could provide stable and reliable data. However, considering the cost and limited size selection, the bead-modified tip would be the best choice for assessing cell stiffness due to its simple geometry, predictable contact area and contact point, and the selective bead size. Nevertheless, the pyramidal tip is more suitable for identifying the mechanical properties of a single cytoskeleton filament, especially in the periphery of a cell. Finally, we suggest that an AFM user should describe all the indenting parameters in as much detail as possible because differences in cell stiffness might directly result from the indenting force or the approaching velocity [51] but have no relation to the natural features of cells.

The effect of temperature changes on the cellular cytoskeleton and morphology have been widely studied in divergent organisms [52], [53]. Cells were cultured at 37°C based on the optimal temperature of cellular function *in vivo*. Although some AFM manipulations for each condition (approximately 60–70 cells) were usually finished within one hour, we wondered whether the operation temperature would affect the E_eff_ of a cell that is determined by AFM. Furthermore, the temperature is also important for the AFM calibration. An inaccurate temperature setting might result in an incorrect spring constant estimation for the AFM tip, biasing the cell elasticity calculation. Our results indicate that the elasticity of a cell measured at an AFM operating temperature of 37°C is undistinguishable from those at 31°C, regardless of medium type. When the temperature was increased to 43°C, the E_eff_ of NIH3T3 cells were significantly decreased (shown as Fig. 2E). Actually, only the temperatures of 37°C and 43°C were set under the control of BioCell®. The temperature of 31°C represented the room temperature in this study, which has been mostly assumed to be 25°C. To prevent interference during the operation, our AFM system was equipped with an anti-vibration table and a custom-built anti-noise box. This type of closed system not only isolates environmental noise and vibrations but also restricts thermal exchange. Consequently, the operation temperature would be higher than the assumed room temperature due to the heat derived from the light source of the inverted optical microscope. Indeed, the temperature of the medium was quite stable at 31°C as measuring by BioCell® and an external thermocouple. A previous study demonstrated that the elasticity of both the nucleus and the periphery of human alveolar epithelial A549 cells were significantly decreased by decreasing the temperature from the physiological temperature to 21°C or 13°C [35]. However, a similar result did not occur in our study, possibly because the operating temperature was not low enough to affect the enzyme activity and the cell cytoskeleton within such a short operation time. When cells were exposed to a heat stress, such as 43°C, they responded with drastic modifications of the various cytoskeletal networks and by a rapid and selective increase in heat shock protein synthesis [54]. One of the obvious changes is the disorganized actin microfilaments (Fig. 3B, i to l). Such change has been linked to heat shock protein phosphorylation [54]. Accordingly, the E_eff_ and morphology of NIH3T3 cells would be severely affected.

The optimal culture medium is essential for the *in vitro* cell culture of eukaryotic cells. The basal medium provides basal nutrients, and the supplement supplies the growth factors for survival, growth, and division. FBS is the most widely used supplement. In this study, we found that the presence of FBS not only increased the cell stiffness but also facilitated the maintenance of the cytoskeleton integrity in response to a temperature stress (Fig. 3A and 3B). In addition to plenty of growth factors, FBS is rich with fibronectin and lipid mediators, such as lysophosphatidic acid (LPA). Fibronectin facilitates cell spreading by activating a specific integrin receptor, and LPA activates Rho activity. Both factors facilitate the formation of stress fibers [55]. Thus, it was reasonable that FBS maintained the prominent stress fiber formation and higher E_eff_ in NIH3T3 cells. However, FBS should accompany a suitable culture medium to elicit the best effect. Traditionally, sodium bicarbonate is used as the primary buffering component in cell culture media. To maintain an *in vitro* environment at physiological pH, cells were cultured in 5% CO_2_ in a humidified incubator. However, such a system does not allow for AFM operation under atmospheric CO_2_, which can affect cellular function due to extreme fluctuations in pH. To address this concern, we cultured NIH3T3 cells under normal culture conditions and changed the medium to DMEM or CO_2_-IDM just before the AFM assessment. Cells exhibited similar elasticity in whichever medium they were cultured. However, the presence of FBS seems to be critical for the cell stiffness. Without the FBS supplementation, cells became softer. Moreover, the effect of FBS was notable for the maintenance of cell stiffness only in DMEM when cells were cultured at lower temperature (31°C) (see Fig. 2E). These data indicate that CO_2_-IDM might not be a suitable medium for maintaining the physical properties of cells during AFM assessment at room temperature. Thus, it has been suggested that cell lines might require either direct or sequential adaptation to CO_2_-IDM for maximum growth performance [56]. This result could be due to incomplete adaptation to CO_2_-IDM within the short operation time at room temperature.

When adhered to ECM-coated substrates, via integrin-mediated binding, cells exhibit a different cell spreading level, which is accompanied by a change in the structure and composition of the cytoskeleton that could be related to changes in cell stiffness. Proper ECM adhesion provides cells not only with survival cues but also with differentiation cues. The transmission of mechanical signals in a cell is dependent on the interaction of integrins with the ECM. Consequently, activated and clustered integrins lead to the recruitment and organization of several different cytoskeletal proteins and reinforces focal adhesion formation. Moreover, RhoA activity was also suggested to involve the increased activation of stress fibers and FA formation [57]. Nevertheless, cells adhered to PLL-coated or non-coated glass slides, via non-integrin-mediated binding, exhibit different cytoskeleton structures. These cells displayed less spreading and sparse actin stress fibers and focal adhesions. Indeed, our data show that the E_eff_ of cells adhered to glass slides coated with COL I, COL IV, FN, or GEL are significantly higher than those adhered on glass or PLL-coated glass slides [5]. There are four different types of stress fibers in cultured animal cells: dorsal stress fibers, transverse arcs, ventral stress fibers, and perinuclear actin cap bundles [58]. AFM assessments of cell elasticity exert a force via the AFM tip to the cell nucleus portion. Therefore, the significance of the perinuclear actin cap in the physical properties of cells should be further emphasized. The perinuclear actin cap consists of stress fibers positioned above the nucleus. Its key function is to regulate the shape of the nucleus in interphase cells with the aid of linkers of nucleoskeleton and cytoskeleton (LINC) complexes [59]. Furthermore, perinuclear actomyosin fibers might act as mechanotransducers to convey force from the cell environment to the nucleus [60]. Recently, Kim et al. demonstrated the importance of “actin cap associated focal adhesions (ACAFAs)” in actin cap formation and function [61]. Compared with “conventional focal adhesions (CFA)”, ACAFAs are larger, fewer in number, and generally located in the periphery of a cell. Considering these results indicates that it was reasonable for cells that were plated on COL I-, COL IV-, FN-, or GEL-coated glass slides to display more ACAFAs and actin cap formation; both would be beneficial for increasing cell stiffness. Moreover, the formation of the perinuclear actin cap was also modulated by substrate stiffness. Wei et al. showed that the low rigidity of a fibrillar collagen gel downregulated β1 integrin activation and clustering and the Y397 phosphorylation of focal adhesion kinase [62]. Cells cultured on collagen gel did not display much spreading and had few actin stress fibers and focal adhesions. Thus, it is not surprising that cells cultured on collagen gel exhibited actin cap dysfunction and the lower E_eff_ (Fig. 4C and 4D).

The major advantage of using cell culture for biological research is the reproducibility and consistency of the results, which can be achieved from using a batch of clonal cells. However, after a period of continuous growth, the cell characteristics might change and become quite different from those found in the starting population. Finally, most cells underwent replicative senescence due to telomere shortening, a consequence of DNA replication. In addition to functional degeneration, the morphology and physical properties of cells might also change during culture. Berdyyeva et al. (2005) demonstrated that primary human foreskin epithelial cells increased their rigidity with ageing *in vitro* due to a higher density of cytoskeletal fibers [63]. Considering these results indicates that, for data consistency and honest presentation, primary culture cells within early passages should be used. In contrast, pluripotent cells possess unlimited self-renewal potential. Under appropriate culture conditions, embryonic stem cells as well as induced pluripotent stem (iPS) cells can be passaged virtually infinitely without any signs of replicative senescence [64]. However, whether the rigidity of pluripotent cells increases with culture passages as it does for other primary cells remains unknown. We had isolated a unique population of cells, the mouse kidney progenitor cells (MKPC), that behaved in manner consistent with renal stem cells [39]. In addition, these progenitor cells exhibited self-renewal of more than 100 passages without evidence of senescence. The study of MKPC motivated our interest in comparing the elasticity of MKPC from different passages. MKPC exhibited no significant difference in their elasticity until passage 90^th^. The E_eff_ of MKPC at the 90^th^ passage was notably increased, regardless of the COLI or FN coated-substrate (Fig. 5A). Actually, MKPC still retained slow-cycling behavior, which is characteristic of stem cells, in *in vitro* culture. Thus, the change in the morphology and stiffness of MKPC occurred over a long time. “Replicative senescence” is associated with telomere attrition during culture expansion. MKPC exhibited low but detectable telomerase activity that was comparable to that measured in other studies in adult tissue-specific stem cells. It is possible that these adult tissue-specific stem cells take an alternative pathway to maintain genome stability. The study by Koch et al. (2013) provided another mechanism by pluripotent stem cells can avoid replicative senescence [65]. The researchers demonstrated that mesenchymal stromal cells, upon long term culture, displayed senescence that was accompanied by specific senescence-associated DNA methylation changes (SA-DNAm), while pluripotent stem cells were able to escape from senescence-associated DNA methylation changes. Whether MKPC escaped from cellular senescence via a similar mechanism remains for further investigation.

Cell-cell interactions play a critical role in the maintenance and functions of the epithelial monolayer [66]. Many studies demonstrated that cell mechanical properties, specifically traction force generation, depend on the presence of neighbors. Cell-cell interactions increased cell traction forces and therefore facilitated the maintenance of cell tension [67]. In this study, we are interested in whether cell-cell interactions affect the mechanical properties of cells. NIH3T3 cells displayed similar elasticity for all densities at which they were cultured. Naturally, NIH3T3 cells rarely formed cell-cell interactions. The signals derived from cell substrate interactions determined the stress formation that contributed to cellular physical properties. In contrast, MDCK cells became stiffer at the confluent density. Both cell-substrate interactions and cell-cell interactions have been known to affect epithelial cell function and morphology. Although cell-cell interactions occurred in MDCK cells regardless of the culture density, their maturations were somewhat different. In the confluent stage, cells within the epithelial monolayer were arranged compactly and accompanied by mature junctions (Fig. 6D). The formation of mature cell junctions consequently strengthened the junctional actin assembly. Furthermore, confluency also modulated the epithelial cell differentiation phenotype. In the confluent stage, MDCK cells showed dense and short microvilli in the apical domain, as confirmed by incrassate apical actin filaments (Fig. S1B). Therefore, we suggest incrassate apical actin and junctional actin dominate the increase in the elasticity of cells cultured in the confluent stage.

In summary, AFM indentation provides a method for revealing cell mechanics by deforming the cell to assess cell elasticity. Our results reveal the importance of using a suitable AFM tip geometry, indenting force at the same loading rate, operating temperature, ECM-coated substrates, and cell density. In addition, selecting an appropriate cell type is also critical for AFM users. Different cell types might display completely different behavior during assessments in the same condition. However, AFM experiments with comparable parameters should be conducted for comparing AFM results from cells in different conditions, pathological levels, or drug treatment.

## Supporting Information

Figure S1 **The effect of plating density on the effective Young’s moduli of cells.** (A) MDCK cells were plated onto COL I-coated glass slides at densities of 5, 50, or 500 cells/mm^2^ overnight. The effective Young’s moduli (E_eff_) of cells were assessed by Bio-AFM. The data were selectively collected by the number of cells that were contacted (1: single surrounding cell; 2: two surrounding cells; 3: three and above surrounding cells). The results were expressed as the mean ± SEM by scatter dot plot. Gray symbols represent the detailed experimental data. (N.S, no significance) (B) Side view of confocal immunofluorescence images of F-actin (red), α-tubulin (green), and the nucleus (blue) from stained MDCK cells that were cultured at densities of 5, 50, or 500 cells/mm^2^, respectively. (Scale bar = 10 µm).
(TIF)

Figure S2 **The AFM images and immunofluorescence image of NIH3T3 cells and 7-4 cells.** NIH3T3 cells and 7-4 cells were plated onto type I collagen-coated glass slides and cultured in culturing medium at a density of 5 cells/mm^2^. The deflection images of AFM were obtained in the contact mode. The scanning rate was set at 200 µm/sec, and proportional and integral gains were instantly adjusted for the cell condition. In general, a single AFM image required approximately 6 minutes. Confocal immunofluorescence images of F-actin (red) staining of NIH3T3 cells and 7-4 cells that were cultured at a density of 5 cells/mm^2^. (Scale bar = 10 µm.).
(TIF)

Figure S3 **The pyramidal AFM tip penetrates the cell membrane.** NIH3T3 cells were plated onto COL I-coated glass slides and cultured in culturing medium at a density of 5 cells/mm^2^. For the pyramidal tip, a 2 nN indenting force and 1 µm/sec approaching velocity was used to indent NIH3T3 cells. An arrowhead was used to indicate the abrupt peak of the force-indentation curve which was resulted from the penetration of pyramidal tip into cell membrane.
(TIF)
