# Supplementary figures and images for: The Influence of Physical and Physiological Cues on Atomic Force Microscopy-Based Cell Stiffness Assessment
Source: PLoS One. 2013 Oct 23;8(10):e77384. doi: 10.1371/journal.pone.0077384 (PMC3806741; doi:10.1371/journal.pone.0077384)

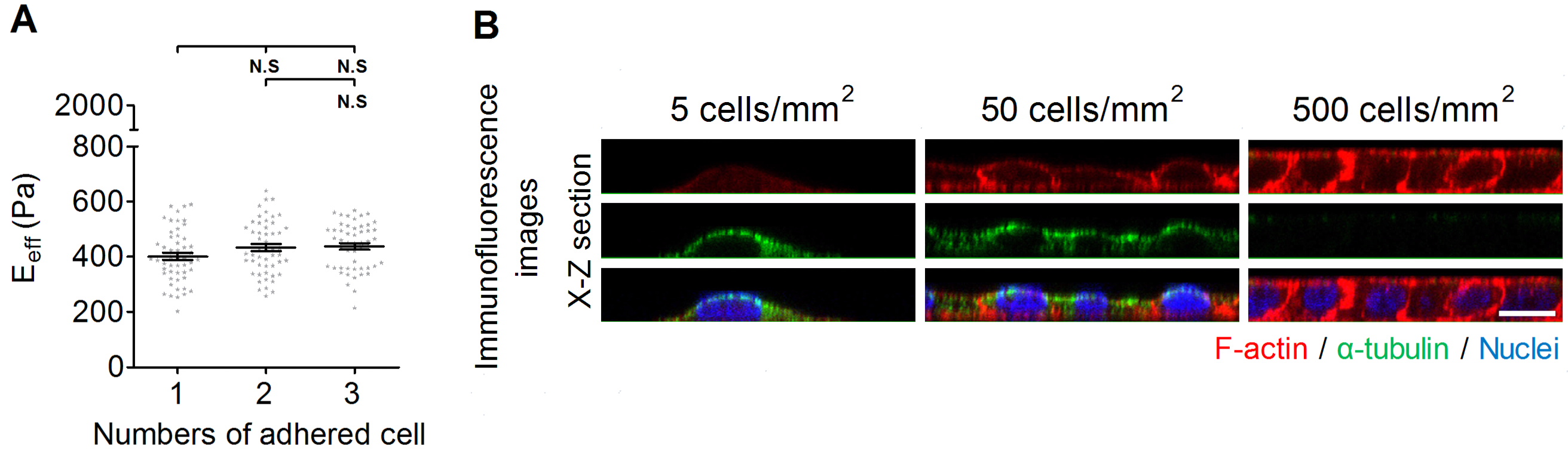

Supplement: Figure S1 — The effect of plating density on the effective Young’s moduli of cells. (A) MDCK cells were plated onto COL I-coated glass slides at densities of 5, 50, or 500 cells/mm2 overnight. The effective Young’s moduli (Eeff) of cells were assessed by Bio-AFM. The data were selectively collected by the number of cells that were contacted (1: single surrounding cell; 2: two surrounding cells; 3: three and above surrounding cells). The results were expressed as the mean ± SEM by scatter dot plot. Gray symbols represent the detailed experimental data. (N.S, no significance) (B) Side view of confocal immunofluorescence images of F-actin (red), α-tubulin (green), and the nucleus (blue) from stained MDCK cells that were cultured at densities of 5, 50, or 500 cells/mm2, respectively. (Scale bar = 10 µm). (TIF) [file pone.0077384.s001.tif]

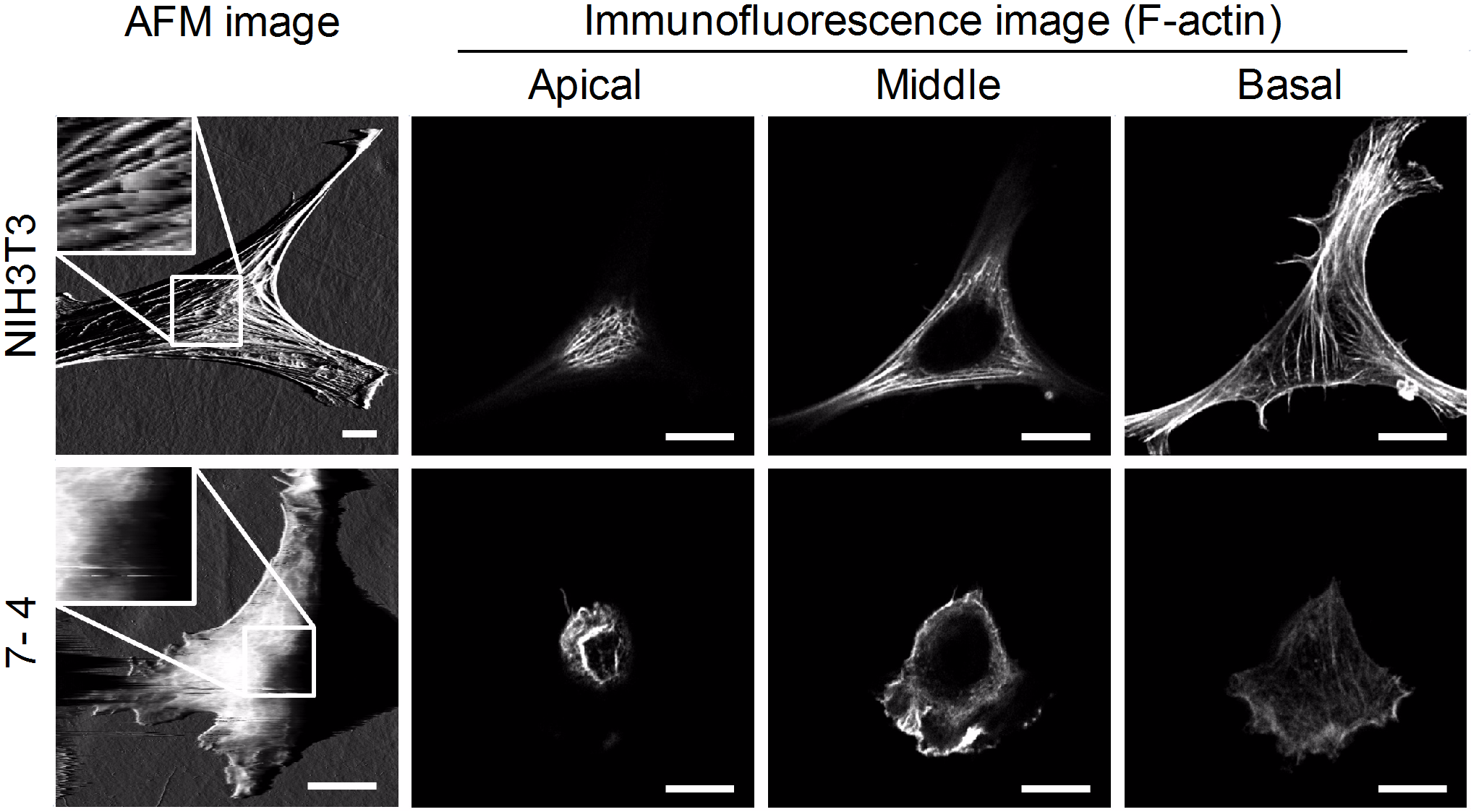

Supplement: Figure S2 — The AFM images and immunofluorescence image of NIH3T3 cells and 7-4 cells. NIH3T3 cells and 7-4 cells were plated onto type I collagen-coated glass slides and cultured in culturing medium at a density of 5 cells/mm2. The deflection images of AFM were obtained in the contact mode. The scanning rate was set at 200 µm/sec, and proportional and integral gains were instantly adjusted for the cell condition. In general, a single AFM image required approximately 6 minutes. Confocal immunofluorescence images of F-actin (red) staining of NIH3T3 cells and 7-4 cells that were cultured at a density of 5 cells/mm2. (Scale bar = 10 µm.). (TIF) [file pone.0077384.s002.tif]

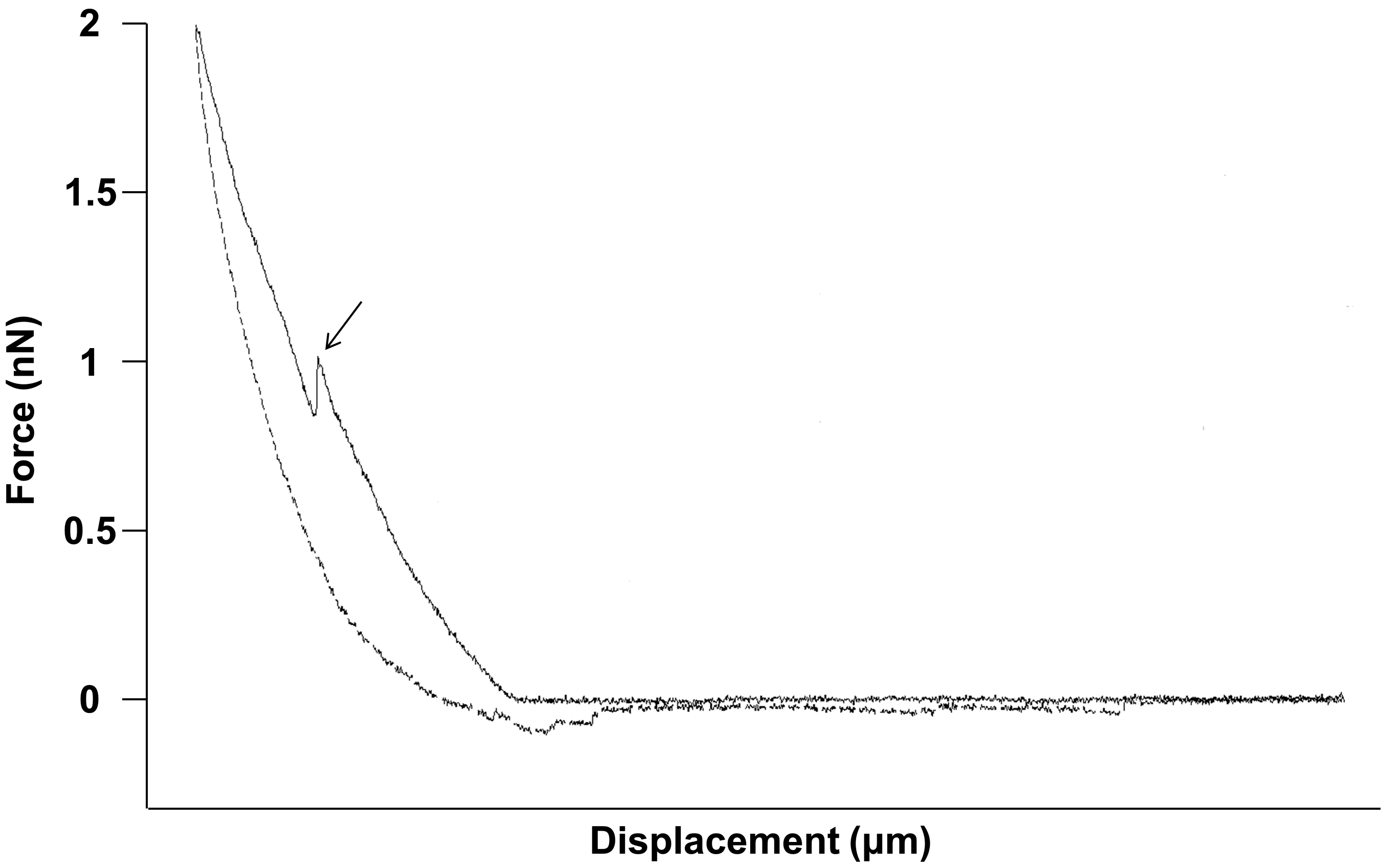

Supplement: Figure S3 — The pyramidal AFM tip penetrates the cell membrane. NIH3T3 cells were plated onto COL I-coated glass slides and cultured in culturing medium at a density of 5 cells/mm2. For the pyramidal tip, a 2 nN indenting force and 1 µm/sec approaching velocity was used to indent NIH3T3 cells. An arrowhead was used to indicate the abrupt peak of the force-indentation curve which was resulted from the penetration of pyramidal tip into cell membrane. (TIF) [file pone.0077384.s003.tif]
